# Pungency Perception and the Interaction with Basic Taste Sensations: An Overview

**DOI:** 10.3390/foods12122317

**Published:** 2023-06-08

**Authors:** Wei He, Li Liang, Yuyu Zhang

**Affiliations:** 1 Food Laboratory of Zhongyuan, Beijing Technology and Business University, Beijing 100048, China; wh960923@163.com (W.H.); gcfll@126.com (L.L.); 2 Key Laboratory of Flavor Science of China General Chamber of Commerce, Beijing Technology and Business University, Beijing 100048, China

**Keywords:** pungent perception, transient receptor potential (TRP) channels, taste sensations, taste interaction

## Abstract

The perception of pungency can be attributed to the combination of pain and heat, and it has critical impacts on food flavor and food consumption preferences. Many studies have reported a variety of pungent ingredients with different Scoville heat units (SHU), and the mechanism of pungent perception was revealed in vivo and in vitro. The worldwide use of spices containing pungent ingredients has led to an increasing awareness of their effects on basic tastes. However, the interaction between basic tastes and pungency perception based on structure-activity relationship, taste perception mechanism and neurotransmission lacks review and summary, considering its brighter prospects in food flavor. Thus, in this review, common pungency substances and pungency evaluation methods, and the mechanism of pungency perception is presented, and the interaction between basic tastes and pungency perception and the possible factors of their interaction are reviewed in detail. Pungent stimuli are mainly transduced through transient receptor potential vanilloid 1 (TRPV1) and transient receptor potential fixed hormone isoform (TRPA1) activated by stimulants. Using modern detection techniques combined with sensory standards, different substances produce different degrees of pungent stimulation, ranging from 10^4^ to 10^7^ SHU/g. Pungent stimuli can affect taste receptor or channel protein conformation and regulate taste bud cell sensitivity by producing neurotransmission products. The products of neurotransmission and taste receptor cell activation in turn act on taste perception. When there are simultaneous effects of taste perception, pungency stimulation may enhance the perception of salty at a certain concentration, with a mutual inhibition effect with sour, sweet, and bitter taste, while its interaction with umami taste is not obvious. However, due to the complexity of perception and the uncertainty of many perceptual receptors or channels, the current studies of interactions are still controversial. Based on the understanding of the mechanism and influencing factors, the availability of pungency substances is proposed in the perspective of food industry in order to achieve new development.

## 1. Introduction

As an important part of the global food culture, natural spices have always performed irreplaceable roles in the diversity of seasoning in association with a special perception, pungency. Since the identification of capsaicinoids in 1876 [1], pungent stimuli have garnered the significant interest of many researchers. Pungency is not strictly a sense of taste, because it is unlike taste (sour, sweet, bitter, salty, and umami). No taste receptor cells (TRCs) for pungent sensation in the taste buds on the surface of the tongue are known [2]. Hence, taste buds cannot express or recognize pungent stimuli directly or specifically. Traditionally, the pungent sensation has been classified as a purely trigeminal stimulation [3], which is a multimodal chemical sensory stimuli [4]. Elicitation of the pungent sensation essentially involves a combination of pain and heat and is often associated with the sting and irritation associated with burning [5]. Analysis of trigeminal nerve sensitivity expression measurements in humans with chronic use of pungent foods revealed that regular pungent stimulation increases the chemical heat-mediated resistance in the trigeminal nerve. Furthermore, a substantial decrease in burning intensity is also noted with these nerve receptors [6].

The transient receptor potential (”RP) ’hannel family is a superfamily of cationic transmembrane proteins capable of responding to a wide range of sensory stimuli. TRP is expressed in multiple tissues and performs an important role in the transmission of taste and perception of chemical stimuli [7]. In the 1990s, David Julius, an American physiologist specializing in receptor cloning, successfully cloned the capsaicin-specific receptor, TRPV1, in 1997 using chili peppers as an entry point and unexpectedly discovered that the receptor could be activated by physical higher temperature above 43 °C [8]. This discovery presents, for the first time, the role of ion channel receptors in signal transduction between physicochemical stimuli. Natural chemical stimuli, such as capsaicin, and physical stimuli, such as temperature, can be uniformly converted into electrical signals via TRPV1 channels on cell membranes. These findings demonstrate the most fundamental source of somatosensory perception at the molecular level and provide an update on the perception of somatic sensations. In the following two decades, Julius used TRPV1 as the starting point and discovered a variety of TRP channel proteins related to somatosensation. Furthermore, he analyzed the three-dimensional structures of various TRP proteins, including TRPV1, and explored the structural and functional relationships of TRP channel proteins by physiological means, such as gene knockout, providing a theoretical basis for targeted drug development. His pioneering and systematic research has led to him being awarded the Nobel Prize in Physiology or Medicine in 2021. 

Today, the most commonly used measure of heat is the Scoville heat units (SHU), introduced by American chemist Wilbur Scoville in 1912. First, the SHU index is nothing more than a taste test for capsaicin. In the test, a certain amount of capsaicin extract was prepared from a certain pepper, and the extract was diluted with water until the tip of the tongue could not feel pungency; the dilution factor represented the unit of pungency. To unify the standards, the experiment stipulated that one unit of spiciness was equal to the pungency that could be diluted to zero with 50 L of water. As this method is subject to subjective influence, high-performance liquid chromatography (HPLC) was used to analyze the pungency of capsaicinoids. However, because the SHU index has been used for a long time, it is currently common to convert the content of a single class of capsaicinoids measured by HPLC to SHU for examination of the degree of pungency by multiplying the corresponding coefficient to indicate the pungency of each pungent substance, the conversion coefficient was obtained by combining the threshold of substance pungency and gas liquid chromatography (GLC) with accuracy [9]. This method can characterize pungency by quantitative standard, reduce the uncertainty caused by subjective sensory factors, and be more precise and efficient. The SHU coefficients of common capsaicinoids are listed as follows: nordihydrocapsaicin (9.3 × 10^3^), capsaicin (16.1 × 10^3^), dihydrocapsaicin (16.1 × 10^3^), homocapsaicin (6.9 × 10^3^), homodihydrocapsaicin (8.1 × 10^3^), and vanillyl pelargonamide (9.2 × 10^3^). In addition to the traditional methods, the innovation of modern technology has promoted the new development of pungency measurement methods. Reported methods include HPLC [10], gas chromatography [11], spectroscopy [12], and electrochemistry [13], which have reduced the cost of detection, greatly improved the sensitivity of detection, and lowered the detection threshold. As some of the instruments are compact and portable, it provides the possibility for field analysis of pungency measurement [14]. 

Many natural compounds contribute to the pungent sensation. These natural pungent ingredients exist in various plants and can be used to protect against microbial and animal damage. These substances can interact with TRP channels in a covalent or noncovalent manner, causing the body to respond to pungent conditions (Table 1). Pain and heat sensations in the oral cavity attributed to pungency will interact with taste sensations and further affect the mediation and recognition of taste [15]. These substances also have many physiological effects (Table 2). Therefore, pungent ingredients added to food can enhance the taste of food and enrich the taste experience. In recent years, the TRP channels involved in the production of pungent sensations have been investigated in detail, and the contribution of pungent ingredients to taste perception has been identified. This review provides a brief overview of the perception of pungent stimuli and discusses the interactions between pungent stimuli and other taste information.

## 2. Common Pungent Ingredients

### 2.1. Capsaicinoids

Capsaicinoids are among the most important active ingredients in the production of pungent sensations. The genus Capsicum contains capsaicinoids, including capsaicin, dihydrocapsaicin, nordihydrocapsaicin, homocapsaicin, and homodihydrocapsaicin. Capsaicin is the main irritating component found in capsicum plants. It is responsible for almost 90% of capsicum pungency, with dihydrocapsaicin. The basic chemical structure of capsaicinoids consists of an aliphatic hydroxyl group in vanillyl alcohol with a fatty acid. Capsaicin is biologically bound to branched-chain fatty acids via vanillin and has a nonpolar phenolic structure. The benzene ring in the structure is modified by an acyl chain that performs a decisive role in the pungent sensation of capsaicin [19]. Capsaicinoids produce strong burns on any tissue it comes in contact with, and capsaicin’s pungency is evaluated in SHU, with 1 g of capsaicin being equivalent to 1.6 × 10^7^ SHU, which is similar to dihydrocapsaicin. The pungency of nordihydrocapsaicin, homocapsaicin, and homodihydrocapsaicin is significantly lower than that of capsaicin and dihydrocapsaicin, about 9 × 10^6^ SHU [48]. Capsaicinoids act as antioxidants by chelating ferrous ions in the body and scavenging DPPH free radicals. Moreover, capsaicin exerts a significant anti-obesity effect by activating TRPV1 channels to reduce lipid levels in cells and regulate lipid metabolism and glucose homeostasis. Interestingly, high concentrations of capsaicin can induce desensitization of depolarized TRPV1 under sustained use, which has an analgesic effect [19].

### 2.2. Allicin

Allicin (diallyl thiosulfinate), derived from the bulb of *Allium sativum*, is also found in onions and other Liliaceae plants [49]. It is an organosulfur compound formed by the catalytic decomposition of alliin in fresh garlic by alliinase [50] and is the main pungent stimulant component in fresh garlic. It can activate cationic currents in TRPV1 and TRPA1 channels in nociceptive neurons by covalently modifying the cysteine residues on the channels. It immediately produces a strong stimulating effect [51,52]. Furthermore, allicin exhibits excellent membrane permeability and readily interacts with sequestered intracellular sulfhydryl compounds to exert its unique physiological activities. Allicin is a natural broad-spectrum antibiotic that inhibits bacterial growth. It also increases nitric oxide synthase (iNOS) activity and superoxide dismutase (SOD) levels in living organisms, inhibits cholesterol microgel formation, and protects the cardiovascular system and liver cells. The allyl sulfide in allicin also exerts anticancer effects.

### 2.3. Allyl Isothiocyanate (AITC)

AITC is found in cruciferous plants, such as horseradish, broccoli, and mustard [53]. It is derived from the high content of glucosinolates in cruciferous plants and is the main pungent component of mustard and mustard oil. When plant tissues are damaged, glucosinolates are hydrolyzed by myrosinase to form AITC with thiocyanate [54]. In physiological studies, AITC has demonstrated strong antimicrobial and biological activities, especially in the areas of anti-inflammation, DNA repair, and cancer risk reduction at low concentrations, owing to its ability to disrupt the cell membrane of bacteria [55,56].

### 2.4. Piperine

Piperine, also known as 1-piperoylpiperidine, is an alkaloid unique to the piperaceae family and has a strong pungent stimulus (1 × 10^5^ SHU/g). For example, the fruits and roots of plants, such as pepper and longan, are rich in piperine [57]. The structure of piperine contains a replaceable benzene ring side chain and a hexahydropyridine ring. The signal activation and desensitization effects of capsaicin on TRPV1 are stronger than those of capsaicin alone. This finding suggests that piperine could perform a role in the treatment of diseases associated with increased TRPV1 expression, such as inflammatory bowel disease [58]. In addition, piperine has also demonstrated analgesic, blood pressure, vascular cell regulation, anticancer, and anti-inflammatory effects [30].

### 2.5. Gingerols and Derivatives

The pungent flavor of ginger originates from the combination of gingerols and their derivatives. The basic structure is a 3-methoxy-4-hydroxy-phenyl functional group connected to a hydrocarbon chain. The mixture can be divided into six categories according to the different fatty chains to which they are attached, including gingerols, zingerone, shogaols, paradols, gingerdirones, and gingerdiols. Among these, the contents of gingerols and shogaols are more abundant, both of which are the main pungent stimulants of ginger and dried ginger, respectively. Gingerol is formed by dehydration of gingerol at high temperatures and low pH and has higher biological activity than gingerol. The pungency of gingerols and shogaols is 8 × 10^4^ SHU/g and 1.5 × 10^5^ SHU/g, respectively. They are also responsible for the pungent sensation in the body through the activation of TRPV1 and TRPA1 [59]. Furthermore, although zingerone, paradols, gingerdirones, and gingerdiols have not been studied much in terms of pungent stimulation, they are still functional factors of great interest, with anti-inflammatory, anti-viral, antioxidant, antiemetic, and anti-cancer physiological activities [60].

### 2.6. Sanshools

Sanshool, a kind of chain unsaturated aliphatic amide alkaloid, is the main source of prickly ash’s pungency (“Ma” taste). Sanshool is mainly found in the prickly ash peel, followed by the flowers and leaves of the prickly ash peel. Sanshools all have conjugated long chains, active amide structures, and alcoholic hydroxyl functional groups. To date, the structures of six kinds of sanshools have been resolved, including α-sanshool, β-sanshool, hydroxy-α-sanshool, hydroxy-β-sanshool, γ-sanshool, and δ-sanshool. Among them, α-sanshool and γ-sanshool can produce a burning sensation in the mouth, hydroxy-α-sanshool and hydroxy-β-sanshool can produce numbness, and β-sanshool can produce a bitter taste [61]. Hydroxy-α-sanshool is the most abundant irritant in prickly ash and is the main compound that causes a tingling sensation; the activation performance is lower than that of capsaicin and allyl isothiocyanate [62]. In addition to TRP channels, sanshools can interact with a variety of ion channels and receptors, such as the two-pore domain K^+^ and G protein-coupled receptors (GPCRs). This is the basis for the development of multiple drugs with anti-inflammatory and analgesic properties, intestinal protection, gastrointestinal diseases, and type I diabetes [63].

### 2.7. Other Pungent Substances

In addition to pungent substances found in common natural species, they are widely found in herbal medicines. However, owing to the wide variations in the spiciness of herbal medicines, the specific pungency of stimulants in various herbs has not been elucidated.

Dried, nearly ripe fruits of *Euodia rutaecarpa* (Juss.) Benth. are not only used as an unfair condiment but are also used as a traditional Chinese medicine in China. Evodiamine is an active substance of quinazoline carbonyl alkaloid that is extracted from *Euodia rutaecarpa* (Juss.) Benth. The chemical structure of evodiamine contains a pyridine ring and an epoxide ring and an aromatic ring between these two rings. Evodiamine is a novel, non-irritating TRPV1 receptor inhibitor that does not induce a pungent sensation in the body [64].

Cinnamaldehyde is a yellow, viscous liquid that is abundant in plants, such as cinnamon, and is found naturally in essential oils, such as Sri Lankan cinnamon oil, cassia oil, patchouli oil, hyacinth oil, and rose oil. The natural cinnamaldehyde in nature is trans structure; the molecule is an acrolein connected to a phenyl group so that it can be considered as an acrolein derivative. Cinnamaldehyde can activate the TRPA1 receptor and lead to acrid stimulation in the body [65], resulting in physiological effects, such as sterilization, antisepsis, anti-ulcer, antiviral, and anti-obesity activities, and regulation of blood circulation and stasis. Interventional studies involving animals or humans, and other studies that require ethical approval, must list the authority that provided approval and the corresponding ethical approval code.

## 3. Sensory Basis and Transmission of Pungent Sensation

### 3.1. Perceptual Mechanisms Associated with Pungent Sensory Information

**Figure 1:**
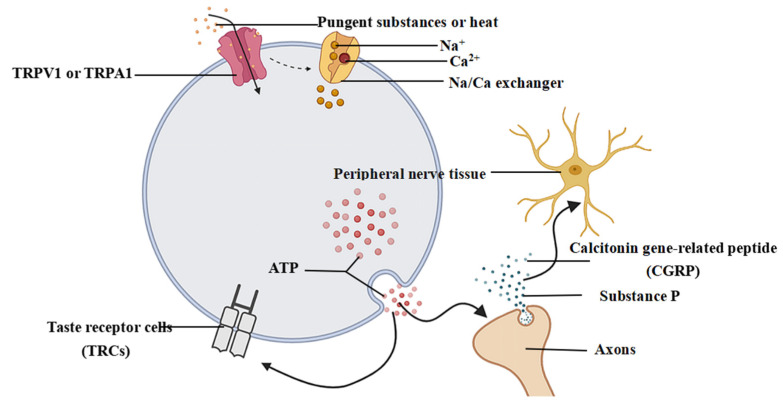
The signal transduction pathway of pungency between TRP channels and nerves.

The trigeminal nerve located on the surface of the mouth and tongue detects stimuli attributed to foreign substances (pain and noxious heat) and produces a relevant response by integrating signals. The two main types of pungent afferent nerves are the Aδ and C nerve fibers, and TRPV1 and TRPVA1 on the fibers are able to bind to pungent substances, which, in turn, are activated and opened [66]. The signal transduction pathway between TRP channels and nerves associated with pungency is shown in Figure 1.

TRPV1, a member of the TRP ion channel family, has been studied extensively as a receptor for most pungent ingredients. TRPV1 receptors are widely distributed in somatosensory neurons and peripheral nerve fibers in the oral and nasal cavities [67] and are mainly expressed in injured neurons in the trigeminal ganglion (TG) and dorsal root ganglion (DRG) [8,68]. TRPV1 can perceive and stably express pain and thermal stimuli and performs an indispensable role in pungent sensations [69]. It is a non-selective functional cation channel [70]. Electron cryomicroscopy analysis showed that the TRPV1 channels have a tetramer structure, and the subunits are arranged in a quadruple symmetric form around the central ion permeation path [71]. Each TRPV1 is composed of six transmembrane proteins (S1-S6) arranged an α-helical structure, and the transmembrane core can be divided into two clusters according to their structure. The transmembrane core is a central ion-conducting pore formed by a tetramer of the S5-P-S6 structural domain that controls the opening and closing of the channel. This channel switch is surrounded by the adjacent S1-S4 voltage-sensing region, thus facilitating the improvement of the binding site of the stimulating ligand to the channel [72]. The ligand-binding pocket of TRPV1 consists of the transmembrane S6-S4 region, the S5-S6 region adjacent to the methyl group, and the S4-S5 region that acts as a linker [73]. The vanillamide structure in capsaicin can selectively activate the TRPV1 channel via chemical thermal stimulation, which causes neurological searing pain and provides the basis for pungent sensation [74]. At the intracellular protein-water interface, the lipophilic tail of capsaicin and the polar region exhibit high affinity for TRPV1 channels. Hence, the binding of capsaicin to TRPV1 is dominated by hydrophobic interactions. Similar to capsaicin, piperine can also act on “vanillic acid receptors” to activate TRPV1 channels, increase the current in the channels at a certain concentration, and promote the dephosphorylation of TRPV1. Activation of TRPV1 by AITC relies on the interaction of AITC at the capsaicin binding site, and activation occurs accordingly to mediate pungency perception by AITC. The activation of TRPV1 by pyocyanin may not require covalently binding to its intracellular reactive cysteine, because the activation of TRPV1 is not structurally specific, and the specific binding mode needs to be further studied [75]. When pungent substances, such as capsaicin, bind to the transmembrane domain of the TRPV1 channel, the TRPV1 channel is activated and opened, which in turn leads to an influx of sodium or calcium ions, depolarization of nerve cells and generation of action potentials [76]. Sensory afferent axons of the TRPV1 channel transmit chemical messages to the central nervous system (CNS) when stimulator ligands bind to sites of action in the transmembrane domain of the channel. Simultaneously, the opposite axons of the channel reflect stimuli back to the peripheral nerve tissue, releasing glutamate and peptides, mainly substance P and calcitonin gene-related peptide (CGRP) [77]. These substances secreted by axonal reflexes can affect the sensitivity of nerve cells near or within the taste buds, thereby regulating taste responses [78]. In addition, TRPV1 can also respond to temperature stimuli above 73 °C, and the activation of TRPV1 is enhanced when both pungency and temperature stimuli are present [79].

TRPA1 channel is also a member of the TRP ion channel family and is the only member of the TRPA subfamily [74]. The TRPA1 channel, similar to the TRPV1 channel, is present in nociceptive neurons in the TG and DRG. It can cooperate with the TRPV1 channel and is co-expressed in somatosensory cells and other parts of the oral cavity, ensuring that individuals respond accordingly to the stimuli they receive [80,81]. Paulsen et al. [82] determined the structure of human TRPA1 using single-particle electron cryomicroscopy. Structurally, TRPA1 has a similar subunit structure to TRPV1. Hence, the TRPA1 subunit is also composed of six α-helical transmembrane proteins (S1-S6), with S1-S7 forming the receptor binding domain and S5 and S6 forming the pore loop of the channel switch. TRPA1 also has a unique structure. At the center of the TRPA1 channel is an α-helix tetramer with polar residues on its surface, which facilitates helix-solvent interactions and mediates subunit assembly of certain TRP heterodimers. The TRPA1 channel is activated by allicin, AITC, and other pungent sensory substances, with allicin being the most potent activator of TRPA1. TRPA1 is at least ten times more sensitive to allicin than the TRPV1 channel [83]. TRPA1 channel activators can be classified into electrophilic and nonelectrophilic categories. The former can activate TRPA1 by binding cysteine or lysine at the cytoplasmic amino terminus as a covalent bond, whereas non-electrophilic activators can activate TRPA1 via non-covalent interactions with the S5-S6 region of the TRPA1 channel and by binding to the transmembrane structural domain [84,85]. For TRPA1, AITC can activate it by covalently binding to cysteine residues within the channel [86]. Furthermore, the TRPA1 response induced by sanshools may involve the interaction between covalent and non-covalent binding sites. Gingerols and shogaols specifically upregulate intracellular calcium concentrations in TRPV1 and TRPA1 channels [87] and activate the heterologous expression of TRPV1 and TRPA1 in rat and human cells.

### 3.2. Transmission and Generation of Pungent Sensation

Primary neurons transmitting pungent information from the mouth and face are located in the TG, whose ganglion cells are pseudo-unipolar neurons with specific receptors sensitive to temperature and pain perception.

The central synapses of the ganglion cells constitute the sensory roots of the trigeminal nerve and the fibers that transmit thermal sensations mainly terminate in the spinal nucleus of the trigeminal nerve. The periganglion synapses constitute the three branches of the trigeminal nerve, namely, the ophthalmic meridian, maxillary nerve, and mandibular nerve, which transmit thermal sensory information to the mouth and face [88,89]. Some thermosensitive neurons in the TG respond to thermal stimuli, and thermally stimulated neurons enhance their sensitivity to temperature stimuli [90,91]. When pungent ingredients, such as capsaicin are present in the oral cavity, the number of neurons responding to temperatures ranging from 32–42 °C increases, and the response is enhanced. However, the neurons responding to noxious temperature stimuli above 43 °C decrease their response to temperature stimuli threshold, which further increases the neurons that respond to stimuli in the temperature range of 32–42 °C. The TG is located near the temporal bone on both sides of the skull and is directly connected to the brain stem through the trigeminal nerve. The spinal trigeminal nucleus subnucleus caudalis (Vc) of the spinal nucleus of the trigeminal nerve has unmyelinated C fibers and thinly myelinated Aδ fibers that can transmit temperature and pain sensations perform a role in the transmission of received sensory information. The information of heat and pain perception can enter the secondary neurons through the main trigeminal nucleus, then be transmitted to the thalamus. Finally, the innervation in the ventral posteromedial nucleus (VPM), medial thalamic nucleus (MTN), and parbrachial nucleus (PBN) reaches the primary somatosensory cortex (nasal cavity, lips, mouth, tongue, etc.) [92,93]. Experiments have shown that when capsaicin is applied to the tongue, injected into the upper lip, or intradermally injected into the cheeks of rats, there is a significant activation effect of rat Vc neurons [94,95]. Neurons transmitting pungent sensation information in the superficial layer of the Vc mainly project fibers to the PBN and thalamus, with the majority projecting fibers to the PBN [93,96]. The PBN, which receives Vc fiber projections, can act as a relay station for thermosensory information and continues to send information to brain regions, including the central amygdala nucleus (CeA), bed nucleus of stria terminalis (BNST), ventromedial hypothalamic nucleus (VMH), periaqueductal gray (PAG), paraventricular hypothalamus (PVH), and preoptic area (POA) [97,98,99]. The lateral paranasal nucleus (IPBN) can protect the body from noxious stimuli and mediate the production of corresponding emotional and protective responses in the body. The pathways of the IPBN to the CeA and BNST are involved in the production of sensation-related emotions and memories [100,101,102]. The pathway from the IPBN to the PAG and VMH can induce the strong labor effect of stimulation and drive avoidance behavior caused by injurious thermal stimuli through downward inhibition [98]. The POA, as an integrated site of thermoregulation in the brain, regulates the thermal effect of body homeostasis, and the IPBN-POA pathway participates in post-stimulus thermoregulation after receiving stimulation [103].

## 4. The Interaction between Pungent Sensation and Taste Sensation

### 4.1. The Interaction between Pungent Sensation and Taste Sensation in the Saliva

Saliva, an essential component of the mouth, lubricates and protects the oral mucosa and promotes flavor perception. Saliva is produced cumulatively by several glands. In order to meet the demands of many functions, the composition of saliva is complex. The main components of saliva are 99.5% water, 0.3% protein, and 0.2% inorganic ions and trace elements [104]. The pungent stimuli in the trigeminal nerve elicited by substances, such as capsaicin, stimulate paracellular pathways in the oral salivary glands and increases saliva production [105]. The effect of salivary secretion in response to capsaicin stimulation is significantly stronger than that of the five basic taste sensations, with stimulation lasting for up to six minutes [106]. In turn, a corresponding increase is noted in the salivary flow rate, buffering capacity, and corresponding changes in ionic composition, which influence the perception of taste compounds via the external environment affecting taste receptors [107]. A recent study by Gardner [108] showed that capsaicin increases the number of proteins in saliva, specifically amylase and mucin 10 (MUC10). These proteins can interact with or metabolize taste compounds, thereby affecting the release and perception of taste.

The increase in the salivary flow rate caused by pungent stimuli is one of the most important factors influencing on taste perception. Saliva flow rate is negatively correlated with the perception of salty and sour tastes [109,110]. In addition, an increase in enzymes and proteins in the saliva performs a role in the perception of taste. For example, the protein composition of saliva affects the sensitivity to salty taste perception [111]. The higher the salivary amylase activity, the weaker the sensitivity to sweet taste perception in men [99], and the less favorable the perception of salty taste in starch-thickened foods [112]. The buffering capacity of saliva, zinc ion content, and glutamate concentration also influences the perception of sour, bitter, and umami tastes, respectively [113,114,115].

Thus, the perception of pungent stimuli is highly individual. The flow rate, the composition of saliva and the perceptual threshold of various taste substances all have important effects. Additionally, differences in the external environment and in the physiological state and age of the individual are largely reflected in the perception as well.

### 4.2. Interaction between Pungent Sensation and Various Tastes

#### 4.2.1. Interaction between Pungent Sensation and Salty Sensation

Pungent stimuli inevitably affect basic taste perception. The frequency of pungent food intake affects the perception of taste [116]. The perception of saltiness is mediated by taste receptor cells that form fungiform papillae or foliaceous papillae of taste buds on the anterior-lateral side of the tongue. With the presence of Na^+^ and Cl^-^ in the oral cavity, saltiness is perceived. The mammalian perception of salty taste is mediated via two main types of pathways, amiloride-sensitive and amiloride-insensitive, which are classified according to the sensitivity of the receptor to the inhibitor amiloride. The former is an amiloride-sensitive epithelial sodium channel (ENaC) located in apical cellular TRCs in the front of the tongue [117]. The latter is an amiloride-insensitive salty taste receptor that performs a major role in the perception of salty taste in humans [118,119]. TRPV1t can mediate the amiloride insensitivity pathway, which in turn acts on salty taste perception processes. The channel is non-specific, responding to cations, such as Na^+^, K^+^, and NH_4_^+^ [120]. TRPV1t, a splice variant of the TRPV1 receptor, is similar to TRPV1 and can be activated in response to capsaicin and temperature [121]. These same activators also suggest that salty taste and pungency perceptions are closely related. Low concentrations of capsaicin promoted the perception of saltiness in rats, whereas high concentrations inhibited the perception of saltiness. The combined effect of capsaicin and piperonyl oleoresin had a significant enhancement of salty taste perception in a NaCl model, effectively reducing NaCl concentrations in medium to high NaCl solutions under the same salinity conditions [64]. When the pungent ingredients are applied to TRP channels in the mouth and tongue, amiloride-insensitive nerve fibers in the chorda tympani will change their response to NaCl in a dose-dependent manner. Due to the consistency of the receptors, low concentrations of substances, such as capsaicin, can activate the receptors instead of cations, thereby reducing the threshold of salty taste perception up to a certain degree. Once the concentration of a pungent substance, such as capsaicin, is too high, the heat and pain caused by the pungency can mask the perception of the salty taste. In addition to ENaC and TRPV1, mTMC4 is a novel voltage-dependent chloride channel associated with the salty taste produced attributed to high salt concentrations. mTMC4-mediated Cl^-^ currents accelerate the cycling of action potentials. This is a novel finding depicting Cl^-^ involvement in salt taste perception [122]. Whether pungent stimuli affect the perception of salty taste through this channel has not yet been elucidated. However, if the TMC4 receptor can be activated by pungent ingredients, the salty taste perception channel with a high salt concentration can be activated using a low salt concentration, which may provide a new theoretical support for salt reduction and salinity enhancement in the food industry. CGRP released by TG also acts on salty taste receptor cells expressed in the amiloride insensitive pathway and affects information transmission associated with saltiness [123,124]. At the central nervous system level, the central gustatory system and mesolimbic structure are closely linked with the presentation of salty taste, and they perform important roles in taste signal processing and mediating hedonic response to taste [125]. The secondary taste cortex, the orbitofrontal cortex (OFC), is involved in the subjective pleasure of taste [126]. The insular cortex and the OFC encode the intensity and pleasure of salty taste information, respectively [127]. The presence of pungent ingredients can modulate brain metabolic activity associated with the salty taste. Low concentrations of capsaicin significantly increase the activity of the insula and OFC when stimulated by salty information at high salt concentrations, and the brain regions activated by pungent and salty information overlapped. This suggests that pungent ingredients can enhance the intensity of salty taste by activating areas of the brain associated with the pleasurable experience of salty substances and increasing the excitability of the OFC nerves [128].

However, due to the diversity of salty taste perception pathways and salty stimuli in humans, it is difficult for the current study to clearly explain the mechanism of salty taste perception in humans. In addition, the neurotransmission process among individuals is highly likely to deviate from the theoretical process due to physiological, psychological, and life environment changes. Thus, the interaction of pungent and saltness perception still needs to be studied more thoroughly.

#### 4.2.2. Interaction between Pungent Sensation and Umami, Sweet, and Bitter Sensation

The receptors for umami, sweet, and bitter taste sensations are all located in type II cells. Sweet and umami senses are expressed by taste receptor type 1 (T1R) GPCRs T1R1/T1R2 and T1R1/T1R3, respectively, and umami taste perception can also be transduced through metabolic glutamate receptors. Similar to T1R1/T1R3, metabolic glutamate receptor also is located on type II taste cells (receptor taste cells) of the taste buds and selectively respond to umami substances, such as sodium glutamate, aspartic acid, and umami peptides [129,130,131]. However, the bitter taste is expressed by taste receptor type 2 (T2R) GPCRs. Following receptor activation, the signal transduction of the three tastes shares an intracellular mechanism [132]. Therefore, the three tastes are very similar in the process of taste interactions. Oral administration of capsaicin and its analogues reduces the perception of quinine (bitter taste) and high concentrations sucrose (sweet taste) [133]. In addition, sweet and bitter tastes mitigate oral burning caused by capsaicin, piperine, and other pungent substances [134]. Bitter substances can reduce the irritation associated with pungent ingredients on the tongue and mouth and have the least effect on the perception of capsaicin compared to substances associated with other tastes. Higher concentrations of capsaicin reduce the perception of bitterness to a greater extent [135]. Umami, which has a weaker interaction than with sweet and bitter, is largely unaffected by heat, capsaicin, or both. These findings suggest the presence of interaction between pungency perception and taste receptor cells in type II cells.

In response to the stimulation of taste information, taste receptor cells release ATP via membrane channels. ATP acts as a transmitter for afferent nerves to stimulate information transmission [136]. Stimulation of the oral cavity and peripheral nerves by pungent ingredients increases the temperature of peripheral nerve sensors, which in turn increases the current in the TRP-melastatin 5 (TRPM5) pathway. This is responsible for the transduction of bitter, umami, and sweet tastes [137]. Thus, the transduction of GPCR-related tastes (sweet, bitter, and umami) increases the sensitivity to temperature [138]. However, presynaptic taste cells also increase 5-hydroxytryptamine (5-HT) excitation [139]. An increase in 5-HT levels causes receptor cells to receive negative paracrine feedback, which diminishes the availability of calcium ions to mediate taste production in taste cells and reduces ATP secretion. Therefore, 5-HT can mediate inhibitory feedback in receptor (II) cells [140]. Substance P produced by pungent ingredients stimulated nerves and performs an important role in umami conduction [141]. Substance P activates the tachyhormone NK1 receptors on umami-sensitive cells, thereby enhancing the umami taste response and compensating for the effects of 5-HT. Under the dual effects of the 5-HT and substance P, the effect of the pungent ingredients on umami expression is not an apparent. In the perception of sweet taste, in addition to natural sweet substances, such as sucrose, artificial sweeteners (AS) are becoming increasingly important in the food industry as dietary supplements. Low concentrations of AS can activate the T1R2+T2R3 sweet taste receptors [37], and with an increase in AS concentration, the bitter taste perception receptors are activated [142]. Similar to capsaicin, AS reduces the activation temperature of TRPV1 and increases the sensitivity of TRPV1 receptors to heat and acids. In addition, sweeteners such as saccharin or aspartame can induce calcium transients in the DRG. Capsaicin-sensitive neurons can be activated by the sweeteners in response [143].

Although the receptors for the perception of sweet, bitter and umami taste are relatively well defined, the sites where the taste substances bind to the receptors are still uncertain in practical studies. It is also unknown whether the simultaneous action of pungent stimuli and taste substances will influence the structural domain conformation of the receptors. This also makes the transmembrane state perception studies of the same type of contradictory in their results.

#### 4.2.3. Interaction between Pungent Sensation and Sour Sensation

The perception of sour taste can prevent the human body from consuming too much dietary acid and maintain the acid-base balance of the system [144] and can also act as a warning of food deterioration. Sour taste receptors in mammals include internal proton-and hydrogen ion-gated channels. Acid stimulation produces internal currents [145], and the internal proton channel refers to the internal proton current channel via which protons pass through the oral space, such as the ENaC channel [146]. Hydrogen ion-gated channels include the apical potassium channel of the basolateral membrane, nonspecific MDEG1 channel of the ENaC/Deg family, and hyperpolarization-activated circular nucleotide-gated cation channel (HCN) [147]. By studying the cross-action between pungent and sour stimuli, it can be concluded that taste buds treated with capsaicin have a strong desensitization effect on the perception of citric acid stimuli. Desensitization can eliminate sour stimuli and reduce the perception of the sour taste, particularly when the concentration of capsaicin is high. Moreover, the perception of pungent stimuli and the perception of sour taste in the oral are inhibited by each other [148]. The TRP channel, which is one of the acid-sensing receptors, has an influx current stimulated by acid stimulation [145]. Sour taste stimulation of the mouth caused by protons and protonates can lead to intracellular acidification in all taste bud cells [149,150]. The penetration of hydrogen ions between the inside and outside of taste bud cells makes the intracellular pH change with the extracellular environment, thus contributing to the conduction of sour taste. The overlap between the perceptual channels of pungent and sour stimuli makes the interaction between the two relatively close, and some responses cannot be easily delineated.

### 4.3. Interaction between Pungent Sensation and Taste Sensation in the Peripheral and Central Nerves

In the periphery, pungent ingredients can stimulate different taste receptors in the gustatory cells. CGRP, one of the main releasers after the activation of the TRPV1 and TRPA1 channels, is essentially a vasodilator neuropeptide consisting of 37 amino acids [151]. CGRP can convey chemical, temperature, and pain stimuli in sensitive mucous membranes of the oral and nasal cavities and performs a role as an efferent transmitter of peripheral taste organs [76,152]. A large number of fine nerve fibers strongly immunoreactive to CGRP have been found in taste buds or connective tissue membranes, which enable wide distribution of the intense electron-dense precipitationin the cytoplasm of neurites, stimulated the production of phospholipase C-inositol triphosphate-mediated transient changes in calcium in type II and III taste. The release of CGRP can also increase the serotonin transmitter necessary for taste transmission [153], which indirectly inhibits the secretion of the taste receptor excitatory transmitter ATP [154]. In addition, spiciness affects the amiloride-insensitive nerve fibers, thereby affecting the response to saltiness [155]. TRPV1 channels can also transmit sour taste information by being activated by sour substances [156].

**Figure 2:**
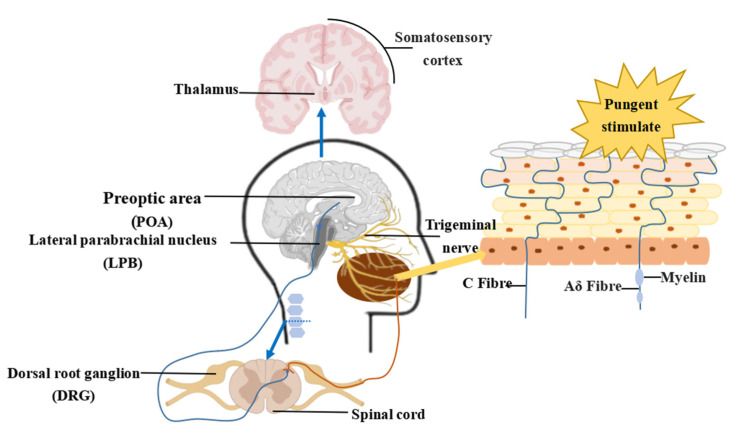
A roadmap for the perception of pungency in the central and peripheral nervous.

Under conditions involving innervation by the cord and facial nerves, taste information can be transmitted to the central nervous system’s transit station, the nucleus tractus solitarius (NTS). The NTS can also receive information from the somatosensory input of the TG nervous system. After integration, it is transmitted to forebrain structures via afferent nerve fibers. Thus, the existence of fiber connections between the TG, NTS, and forebrain provides the morphological basis for the interaction between pungent and taste sensations in the central nervous system [157,158]. The CeA, lateral hypothalamus, and gustatory cortex (GC) in the forebrain region can generate electrical stimuli to regulate NTS neurons, thereby activating neurons in IPBN that respond to different taste sensations [159]. IPBN can also project to CeA via CGRP-containing neurons and experience aversive taste stimuli and nociceptive pungent stimuli [160,161]. This indicates that the aversive taste information encoded in IPBN, such as bitter, sour, and salty sensations, has regional overlap with the pungent sensation, and that the differences in encoding may be attributed to differences in temporal and spatial patterns (Figure 2).

In summary, pungent ingredients in food can affect consumers both physically and emotionally. Additionally, food companies can add flavor dimensions and layers to their products based on taste, smell, and texture through the application of pungency ingredients. In addition, studies have shown that people who regularly consume pungent foods are more motivated in their pursuit of diversity [162]. The curiosity seeking is also one of the potential markets that companies can tap into. For the most important aspect of taste, those who consume pungent ingredients infrequently perceive that the stimulating nature of pungent ingredients affects the palatability of food, whereas those who often eat pungent foods believe that the presence of pungent stimuli can enhance the taste. This is an important way that food companies can use to improve their products. For example, pungent stimulation can improve the perception of salty and sour taste in the mouth to a certain extent. This is a good opportunity for high-salt food companies to adapt themselves to the trend of low-salt healthy diet.

## 5. Conclusions and Future Perspectives

This review systematically summarized and discussed the interaction between the perception of pungency and basic tastes. It includes common natural pungency ingredients, the perception and transmission of pungency, and the influence and possible mechanism of the perception of pungent and basic tastes. Pungency stimuli are perceived through TRPV1 and TRPA1 channels. Stimuli generally contain vanillyl alcohol, amides and other similar structures that activate channel proteins and cause cationic current effects. The neurotransmission induced by channel activation secretes substance P and CGPR, which modulate the sensitivity of nerves near the taste buds and thus affect taste perception at the neural level. In addition, pungency stimuli affect the physiological state of saliva and various taste receptors in the oral cavity. Pungency substances enhance saltiness perception at low concentrations and have a reciprocal inhibitory effect with sweet and bitter taste. The ATP produced by TRCs, and 5-HT produced by synaptic cells have feedback regulation on umami perception together with substance P, making the effect of pungent stimuli on umami perception insignificant. Due to the overlap of pungency and sour taste perception channels, there is mutual inhibition between them, and they can be desensitized quickly. It is worth mentioning that since pungency perception involves the action of multiple neurons, there is also a correlation between its intake and the psycho-emotional state of the person consuming it. This may add reverie to the development of food quality in multiple dimensions.

At present, the study of the interactions between pungency taste sensations and their mechanisms still needs to be explored in depth, and the existing research is not sufficiently systematic, which needs to be further studied at the organism, cellular, and genetic levels. The diversity of pungent substances and the structural complexity of TRPs and taste receptors also render the study of the mechanism of their interaction difficult. In terms of practical application, the use of pungent components affects the taste perception threshold, changing the sensitivity of taste bud cells to various tastes for enhancing, masking, or inhibiting other tastes. For example, if pungent ingredients are used to enhance salty taste perception and reduce the salt content in food industry products, the food industry will more cater to the concept of healthy eating. This study also provides new ideas for the development of palatable products in the food industry. However, the dose and characteristics of these pungent substances remain unclear, and their safety needs to be considered in clinical practice.

## Figures and Tables

**Table 1:** Names, sources, and properties of natural pungent ingredients.

Natural Pungent Ingredients	Main Ingredients	Molecular Formula	Chemical Formula	CAS#	Threshold Pungency (10^5^ SHU)	Natural Sources
Capsaicinoids	Capsaicin	C_18_H_27_NO_3_	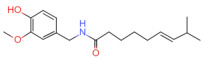	404-86-4	160	*Capsicum annuum *L. (e.g., chili peppers)
	Dihydrocapsaicin	C_18_H_29_NO_3_	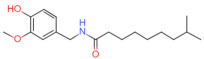	19408-84-5	160	
	Nordihydrocapsaicin	C_17_H_27_NO_3_	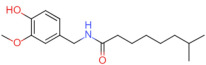	28789-35-7	91	
	Homocapsaicin	C_19_H_29_NO_3_	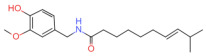	58493-48-4	86	
	Homodihydrocapsaicin	C_19_H_31_NO_3_	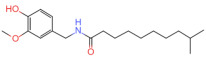	20279-06-5	86	
Piperine	Piperine	C_17_H_19_NO_3_	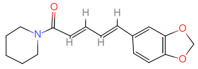	94-62-2	1.0	*Piperaceae* Giseke.
Allyl isothiocyanate	Allyl isothiocyanate	C_4_H_5_NS	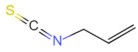	57-06-7	-	*Brassicaceae* Burnett. (e.g., mustard, wasabi, kohlrabi, radish)
Allicin	Allicin	C_6_H_10_OS_2_	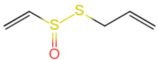	539-86-6	-	*Liliaceae* Juss. (e.g., garlic, onion)

**Table 2:** Physiological effects of common natural pungency ingredients in recent research.

Natural Pungent Ingredients	Physiological Effects	Experimental Phenomenon	References
Capsaicin	Antioxidant	In vitro: reduced lipoxygenase activity and lipid peroxidation significantly. Animal experiment: The oxidative stress levels in the liver and testis of SD rats were significantly decreased, and the contents of GSH-Px and GSH were significantly increased.	[16,17]
	Anti-obesity	Reduced neutral fat content, fat accumulation, lipid droplet size, and surface area; Improved the release of glucagon and the absorption of glucose in the gastrointestinal tract; Improved postprandial hyperglycemia and hyperinsulinemia and fasting lipid metabolic disorders in women with GDM, reduced the incidence of LGA newborns.	[18,19]
	Analgesic	Relieved knee osteoarthritis pain, fibromyalgia, and postherpetic neuralgia.	[20]
	Anti-cardiovascular and cerebrovascular diseases	Significant neuroprotective effect on hypoxic neuron model in vitro and cardiac arrest model in vivo.	[21]
	Anti-inflammatory	Alleviated the inflammation response and the Warburg effect in a TRPV1-independent manner by targeting PKM2-LDHA and COX-2 in sepsis.	[22]
Dihydrocapsaicin	Anti-cardiovascular and cerebrovascular diseases	Protective mechanisms of brain injury after cardiac arrest and resuscitation; Markedly abrogated TNFα-induced expression of the adhesion molecules VCAM-1 and ICAM-1, IL-6 production, and activation of NFκB, Reduced inflammatory damage in human vascular endothelial cell cultures.	[23,24]
Piperine	Anticancer	Inhibited the epithelial-mesenchymal transition (EMT) activated by TGF-β and prevented the invasion and metastasis of HepG2 cells in hepatocellular carcinoma.	[25]
	Anti-inflammatory	Enhanced FAM134B and CCPG1-dependent ER phagocytosis to reduce ER stress, thereby alleviating pancreatitis injury; Repressed CS-induced infiltration of inflammatory cells and thereby exaggerated production of pro-inflammatory mediators and oxidative stress.	[26,27]
	Anti-cardiovascular and cerebrovascular diseases	Improved myocardial ischemia, cardiac injury, and cardiac fibrosis, inhibited vascular smooth muscle cell proliferation, and prevented arterial stenosis.	[28]
	Immunoregulation	Regulated PI3K/AkT-mediated anti-apoptosis signal transduction and improves pancreatic β-cell dysfunction.	[29]
	Antioxidant	Easy to react with high oxidation free radicals, scavenged DPPH, TEMPO, hydrogen peroxide, and reduced Fe^3+^.	[30]
	Anti-obesity	Reversed HFD-induced liver lipid accumulation and insulin resistance via the inactivation of adiponectin-AMPK and PI3K-Akt signaling; Regulated energy homeostasis and inflammation and alleviates obesity associated with GM regulation.	[31,32]
Allyl isothiocyanate	Anticancer	Inhibited Akt/mTOR proliferation signaling and promoted mitochondria-dependent apoptotic pathway through AITC-enhanced activities of caspase-3 and caspase-9 in CAR cells	[33]
	Antibacterial	Prevented *A. niger*, *A. carbonarius* and *A. ochraceus* from infecting grapes and maize and controlled Ochratoxin A contamination; More effective in controlling yeast and Gram-negative bacteria than Gram-positive bacteria.	[34,35]
Allicin	Anticancer	Inhibited the proliferation and promoted apoptosis of various colorectal cancer cells.	[36,37]
	Antibacterial	Inhibited DNA gyrase activity in bacteria and has natural antibacterial properties.	[38]
	Anti-cardiovascular and cerebrovascular diseases	Decreased serum levels of IL-1β, IL-6, and TNF-α, improved calcium homeostasis in cardiomyocytes, and downregulated calcium transport related CaMK II and inflammation related NF-κB and NLRP3, inhibited the activation of CaMK II/NF-κB pathway and protected hypertensive vascular and cardiac remodeling in spontaneously hypertensive rats.	[39]
Gingerols	Immunoregulation	Inhibited viral neuraminidase activity and boosted hemagglutinin-specific CD4 T cell response to the infection; Increased expression of pro-inflammatory cytokines and enhanced Th1/Th17 responses.	[40,41]
	Anti-inflammatory	Attenuated NF-κB/MAPK signaling pathways, formation of ECM, production of inflammatory cytokines, and injury to mammary gland cells both in vivo and in vitro.	[42]
	Antioxidant	Had a high scavenging capacity of DPPH and ATBS radicals, retarded lipid oxidation, and hydrolysis.	[43]
	Anti-obesity	Inhibited adipogenic differentiation and lipid accumulation and activated the Wnt/β-catenin signaling pathway during adipogenic differentiation.	[44]
Sanshools	Antioxidant	Ameliorated spontaneous locomotion deficit of mice induced by D-galactose (D-gal) and AlCl_3_ treatment, reduced malondialdehyde production, and increased the activity of antioxidative enzymes, showing an inhibitory effect on oxidative stress.	[45]
Evodiamine	Anticancer	Induced M-phase cell-cycle arrest by inactivation of CUL4A E3 ligase, and suppressed the growth of esophageal squamous cell carcinoma both in vitro and in vivo.	[46]
Cinnamaldehyde	Antibacterial	Inhibited the growth of an array of microorganisms such as bacteria, molds, and yeasts, inhibited toxin production by micro-organisms.	[47]

## Data Availability

Data is contained within the article.
